# Transcriptome sequencing analysis of the role of miR-499-5p and *SOX6* in chicken skeletal myofiber specification

**DOI:** 10.3389/fgene.2022.1008649

**Published:** 2022-09-15

**Authors:** Yi-Fan Liu, Ming Zhang, Yan-Ju Shan, Li-Chuan Pang, Gai-Ge Ji, Xiao-Jun Ju, Yun-Jie Tu, Shi-Ying Shi, Hao Bai, Jian-Min Zou, Jing-Ting Shu

**Affiliations:** ^1^ Jiangsu Institute of Poultry Science Innovation Co., Yangzhou, China; ^2^ Key Laboratory for Poultry Genetics and Breeding of Jiangsu Province, Jiangsu Institute of Poultry Science, Yangzhou, China; ^3^ Joint International Research Laboratory of Agriculture and Agri-Product Safety, The Ministry of China, Yangzhou University, Yangzhou, China

**Keywords:** chicken, meat quality, muscle fiber, RNA sequencing, miR-499-5p, Sox6

## Abstract

MicroRNAs (miRNAs) might play critical roles in skeletal myofiber specification. In a previous study, we found that chicken miR-499-5p is specifically expressed in slow-twitch muscle and that its potential target gene is *SOX6*. In this study, we performed RNA sequencing to investigate the effects of *SOX6* and miR-499-5p on the modulation and regulation of chicken muscle fiber type and its regulatory mechanism. The expression levels of miR-499-5p and *SOX6* demonstrated opposing trends in different skeletal muscles and were associated with muscle fiber type composition. Differential expression analysis revealed that miR-499-5p overexpression led to significant changes in the expression of 297 genes in chicken primary myoblasts (CPMs). Myofiber type-related genes, including *MYH7B* and *CSRP3*, showed expression patterns similar to those in slow-twitch muscle. According to functional enrichment analysis, differentially expressed genes were mostly associated with muscle development and muscle fiber-related processes. *SOX6* was identified as the target gene of miR-499-5p in CPM using target gene mining and luciferase reporter assays. *SOX6* knockdown resulted in upregulation of the slow myosin genes and downregulation of fast myosin genes. Furthermore, protein-protein interaction network analysis revealed that *MYH7B* and *RUNX2* may be the direct targets of *SOX6*. These results indicated that chicken miR-499-5p may promote slow-twitch muscle fiber formation by repressing *SOX6* expression. Our study provides a dataset that can be used as a reference for animal meat quality and human muscle disease studies.

## Introduction

Meat quality is an important livestock economic characteristic and is determined by muscle color, water holding capacity, pH, tenderness, flavor substance content, and intermuscular fat. As an essential tissue accounting for 40% of an animal’s body weight, skeletal muscle contains muscle fibers that demonstrate diverse biochemical and structural features, and different fiber compositions in skeletal muscle are closely associated with muscle quality (Lee et al., 2010; Joo et al., 2013; Ismail and Joo, 2017). Myofibers within chickens are classified as white and red fibers; they indicate glycolytic (type IIB; undergo glycolytic metabolism and rapid contraction) and oxidative (type I/IIA; undergo oxidative metabolism and slow contraction) fibers, respectively (Zierath and Hawley, 2004; Hakamata et al., 2018).

The skeletal muscle fiber type is directly determined by various genetic factors. miRNAs are small (22 nucleotides) non-coding RNAs, which show negative gene regulation at the post-transcription level by combining with the 3'-untranslated region (3'UTR) in target mRNAs (Gladka et al., 2012). Several studies have suggested that miRNAs regulate skeletal muscle function, including fiber-type transition and differentiation (Zhang et al., 2014; Zhang et al., 2019; Yin et al., 2020).

miR-499-5p has been identified as a myosin-encoding miRNA, which is recognized as an essential regulatory factor for muscle fiber conversion and as a muscle disease biomarker (Liu et al., 2016; Wan et al., 2018; Shi et al., 2019). Van Rooij et al. first reported that miR-499-5p and miR-208b knockouts in mice resulted in slow-to-fast alteration of muscle fiber type (van Rooij et al., 2009). In mammals and fish, miR-499 is highly expressed within slow-twitch muscles, which possibly modulates the types of muscle fibers via target mRNA, including *SOX6*, *THRAP1*, *FNIP1*, *ROD1,* and pathways, including NFATc1/MEF2C and FNIP1/AMPK (Nachtigall et al., 2015; Liu et al., 2016; Wang et al., 2017; Xu et al., 2018). Nonetheless, the possible role of miR-499-5p and related mechanisms in chicken muscle fiber switching have not been clarified.

RNA-seq is an effective tool for gene function studies because it can obtain global gene expression levels in tissues or cells with high accuracy (Liu et al., 2017; Pu et al., 2022). In this study, RNA-seq was used to explore the biological function of miR-499-5p in chicken skeletal muscle fiber specification and to identify the target gene of miR-499-5p in chicken primary myoblasts (CPMs). Our findings may enable a better understanding of the underlying mechanisms implicated in the quality performance of chicken meat.

## Materials and methods

### Muscle sampling

The Wenchang chickens used for ATPase staining and quantitative real-time PCR (qRT-PCR) assays were obtained from Tanniu Chicken Co., Ltd. (Haikou, China). Six 20-weeks-old hens of similar body weights (1,315–1,430 g) were slaughtered by electrical stunning and exsanguination. Intermediate section samples of various skeletal muscles were collected after rapid dissection, followed by freezing in liquid nitrogen and preservation at -80°C. Skeletal muscle samples included the *pectoralis major* (PM), *sartorius* (SA), *lateral pectineus* (LP), and *medial gastrocnemius* (MG) muscles.

### Myosin ATPase staining

According to our previous report, myofiber types were identified using myosin ATPase staining (Li et al., 2016). As described by Brooke and Kaiser (1970), type IIB was intensely active and stained dark brown, type IIA was weakly active and stained light brown, and type was inactive and non-stained.

### Cell culture and transfection

We separated CPMs in E11 chicken leg muscles according to a previous description, followed by culturing in DMEM/F12 medium (Gibco, Carlsbad, CA, United States) containing 20% fetal bovine serum (FBS; Gibco) (Shan et al., 2020). When the density reached 80%, FBS was replaced with 5% horse serum (Procell, Wuhan, China) to induce myogenic differentiation. miR-499-5p mimics, NC inhibitor, siRNA targeting *SOX6*, or siRNA negative control were transfected into CPMs using Lipofectamine 3,000 (Invitrogen, Carlsbad, CA, United States) following the manufacturer’s protocol. The miRNA mimics and siRNAs were prepared by Ribobio Co., Ltd (Guangzhou, China). The *SOX6* siRNA sequence was 5'-GGT​GAA​TAC​AAA​CAA​CTG​A-3'. After 48 h of transfection, each cell group with four replicates was collected for the subsequent RNA-seq and qRT-PCR assays.

### RNA extraction and sequencing

Total RNA was extracted from CPMs using TRIzol reagent (Invitrogen, Carlsbad, CA, United States) following the manufacturer’s protocol. An Agilent 2,100 Bioanalyzer (Agilent Technologies, Santa Clara, CA, United States) and a NanoDrop 2000 spectrophotometer (Thermo Fisher Scientific, Waltham, MA, United States) were used to check RNA quality. High-throughput RNA-seq was performed on an Illumina HiSeq 4,000 platform (Illumina, San Diego, CA, United States) at Gene Denovo Biotechnology Co. (Guangzhou, China).

### High-throughput sequencing data analysis

For quality control, fastp v0.18 with default parameters was utilized to process the raw data, followed by alignment of paired-end clean reads against the reference genome (*GRCg6a*) with Hisat2 (Sirén et al., 2014; Chen et al., 2018). FeatureCounts were used to count reads mapped into diverse genes, and fragments per kilobase million (FPKM) values were determined for diverse genes (Liao et al., 2014). Differentially expressed genes (DEGs) in the treatment and control groups were identified using the DESeq2 R package (Love et al., 2014). DEGs were identified based on the threshold of false discovery rate (FDR) < 0.05 with DESeq2. Target genes of miR-499-5p were identified using the TargetScan 7.2 (http://www.targetscan.org/vert_72/) and miRDB (http://mirdb.org/) web platforms with default parameters.

Gene ontology (GO) enrichment analysis of DEGs was performed using an online tool from OMICSHARE (www.omicshare.com/tools). Significant enrichment of GO terms was indicated by *p*-value < 0.05. OMICSHARE was used to identify significantly enriched DEGs in the Kyoto Encyclopedia of Genes and Genomes (KEGG) pathways. The STRING database (https://string-db.org/) was utilized for protein-protein interaction (PPI) network construction at moderate confidence. Cytoscape software v3.5.1 was used for visualization.

### qRT-PCR

The Primer3 website (https://bioinfo.ut.ee/primer3-0.4.0/) and miRprimer2 software (https://sourceforge.net/projects/mirprimer) were used to design qRT-PCR primers for genes and miR-499-5p, respectively. Primer information is listed in Supplementary Table S1. For mRNA quantification, cDNA was prepared using the HiScript III 1st Strand cDNA Synthesis Kit (+gDNA wiper) (R312, Vazyme, Nanjing, China). qRT-PCR with ChamQ SYBR Color qRT-PCR Master Mix (Q411, Vazyme) was performed for cDNA quantification using a real-time PCR system (Mx3000P, Agilent Technologies). For miRNA quantification, cDNA was synthesized using the miRcute Plus miRNA First-Strand cDNA kit (KR211, Tiangen, Beijing, China), and qRT-PCR was performed using miRcute Plus miRNA qPCR kit (SYBR Green) (FP411, Tiangen). Chicken *GAPDH* and U6 snRNA were used as endogenous references. The 2^−ΔΔCt^ method was used to calculate the fold-change.

### Luciferase reporter assay

DF-1 cells were used for the luciferase reporter assay. The PmiR-RB-Report vector was used to synthesize mutant (MUT) and wildtype (WT) luciferase reporter vectors at Ribobio Co., Ltd (Guangzhou, China). The information on mutant sites in the luciferase reporter vectors is presented in Supplementary Table S2 **.** The cells were transfected with MUT or WT constructs and miR-499-5p mimics or NC mimics. After 48 h, luminescence was detected using a Dual-Glo Luciferase Assay System (E2920, Promega, Madison, WI, United States).

### Western blotting

CPMs were seeded in six-well plates and transfected with overexpression miRNA mimics for 48 h. Cells were harvested, washed with 1× phosphate buffered saline (PBS) and lysed in RIPA lysis buffer. Immunoblotting was performed using standard procedures. The primary antibodies used were anti-SOX6 (NBP2-20458, NOVUS, Saint Louis, MI, United States) and anti-GAPDH (ERAB0003, Erwanbiotech, Shanghai, China). The goat anti-rabbit IgG-HRP (111-035-003; Jackson immune Research, Bar Harbor, ME, United States) was used as the secondary antibody.

### Statistical analysis

The results of the fiber type content, miR-499-5p, and gene levels within muscles and cells are presented as mean ± SD. The expression patterns of the genes and miR-499-5p were statistically compared among the different muscle groups using the one-way analysis of variance (ANOVA) followed by Tukey’s test, as implemented in the SPSS Software Suite (SPSS version 20.0). The statistical significance of qRT-PCR analysis results and luciferase reporter assay results between the treatment and control groups was determined using *t*-test. Differences were considered statistically significant at *p*-values of < 0.05 and < 0.01.

## Results

### miR-499-5p and *SOX6* expression levels in different skeletal muscles

**FIGURE 1:**
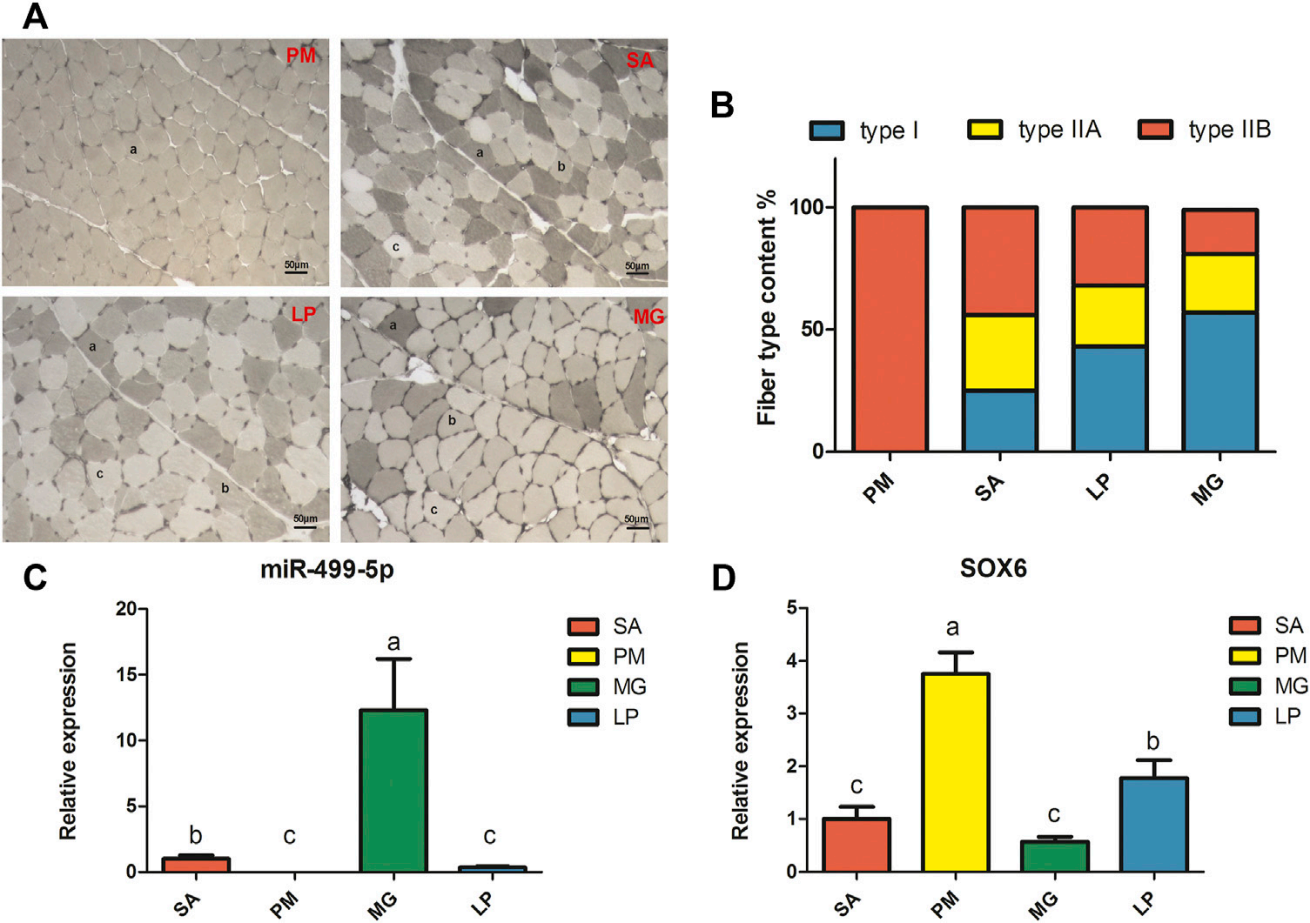
Myofiber type phenotype and gene expression levels in four different skeletal muscles. **(A)** Myosin ATPase staining of four different skeletal muscles. PM: *pectoralis major*, SA: *sartorius*, LP: *lateral pectineus*, MG: *medial gastrocnemius*. **(A)** type IIB fibers, **(B)** type IIA fibers, **(C)** type I fibers. **(B)** Fiber type content of four different skeletal muscles. Relative expression levels of miR-499-5p **(C)** and *SOX6* **(D)** in four different skeletal muscles. All results are shown as mean ± SD. *p* < 0.05 or less is considered significant and indicated with different letters.

As shown in Figures 1A,B, four different skeletal muscles—the PM, SA, LP, and MG, exhibit different muscle fiber compositions. PM is composed entirely of type ΙIB fiber, whereas the other three contain three types of muscle fibers. SA has the highest content of type ΙIA fibers, whereas MG has the most amount of type fiber.

The qRT-PCR results showed that miR-499-5p and *SOX6* have opposing expression patterns. As shown in Figure 1C, miR-499-5p showed the highest expression in MG, followed by SA and LP, and the lowest expression in PM. As expected, *SOX6* was most significantly upregulated in PM, followed by LP, SA, and MG (Figure 1D). The expression level of miR-499-5p was found to have a significant positive correlation with type I fiber content and negative correlation with type IIB fiber content in skeletal muscles, whereas *SOX6* expression showed a similar correlation with type IIB fiber content (Supplementary Table S3).

### Effect of miR-499-5p overexpression on CPM transcriptome

**FIGURE 2:**
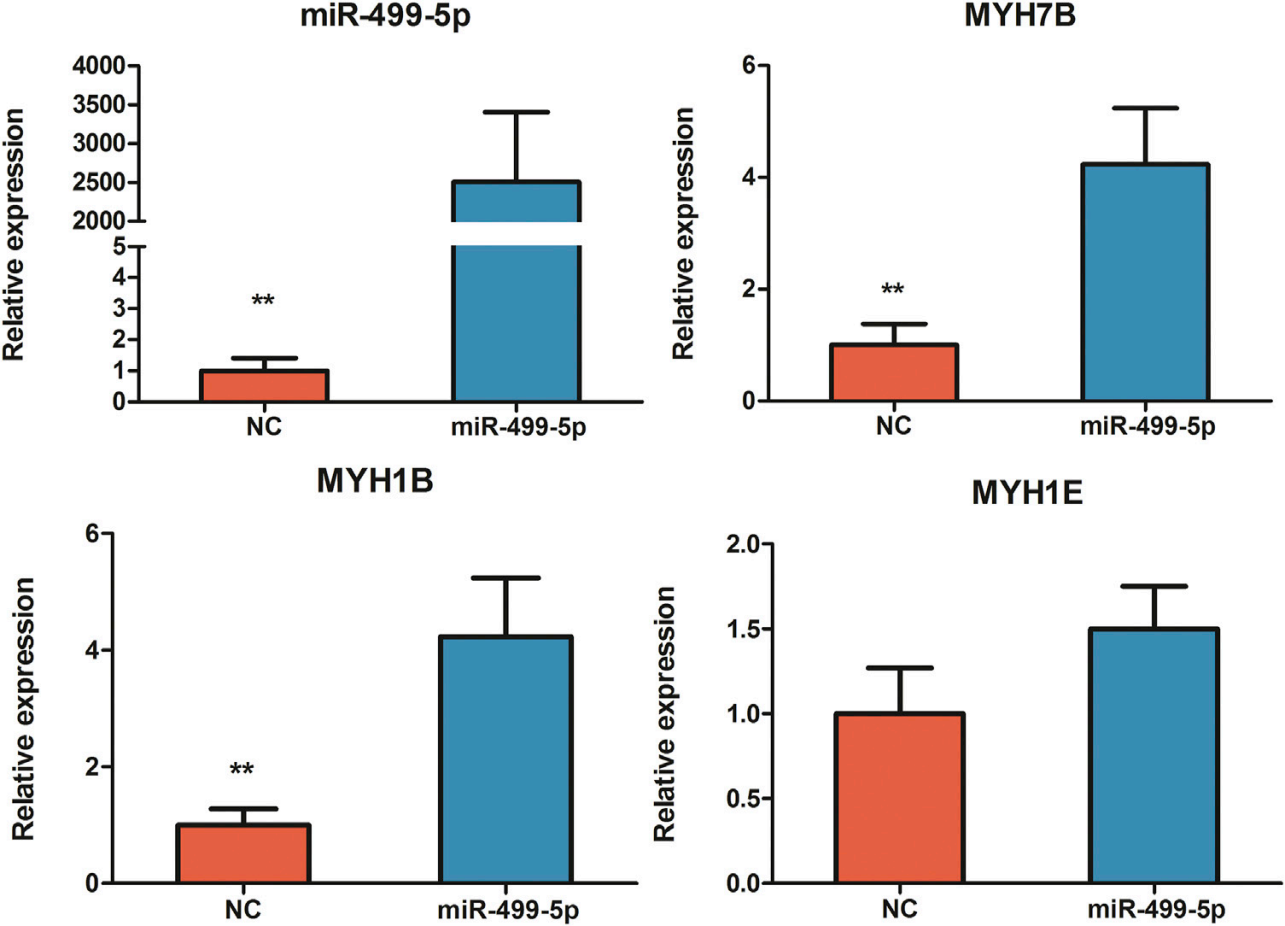
The relative expression levels of miR-499-5p, *MYH7B*, *MYH1B*, and *MYH1E* in miR-499-5p overexpressing CPMs and negative control by qRT-PCR. All results are shown as mean ± SD. **p < 0.01 as compared with the control.

**FIGURE 3:**
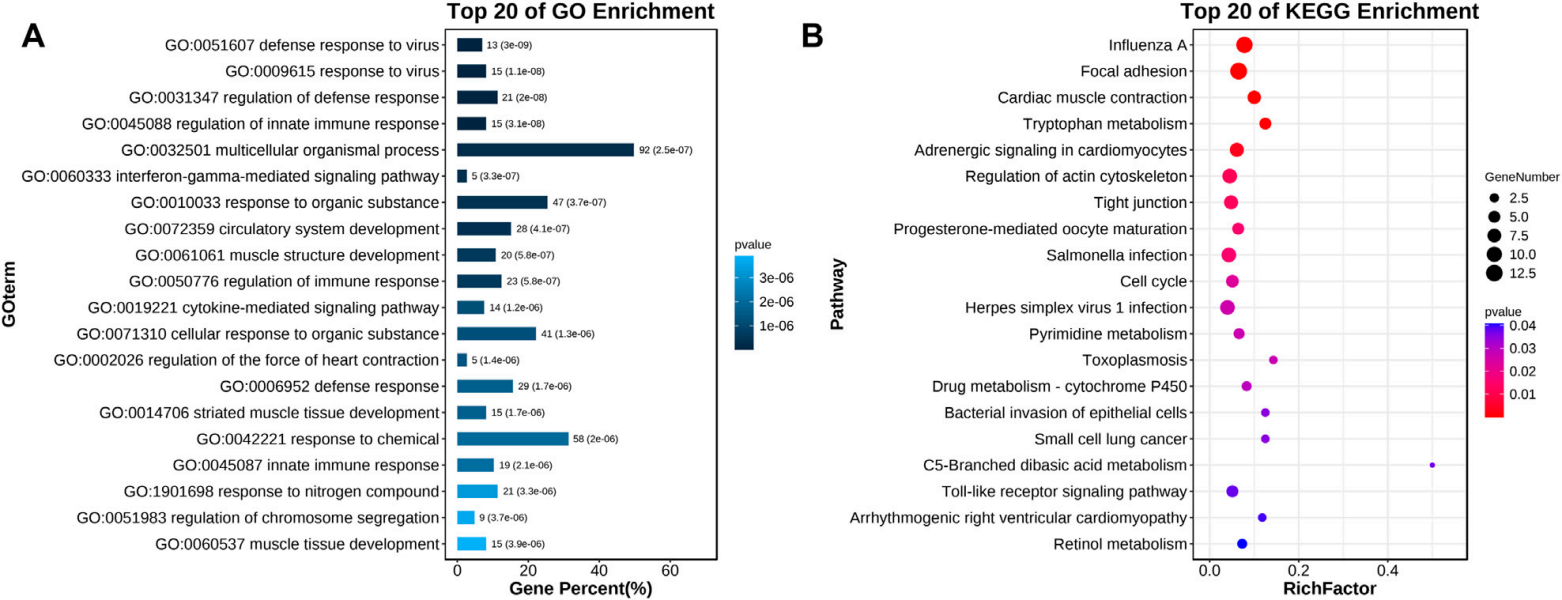
Functional enrichment of DEGs between miR-499-5p overexpressing and NC CPMs. **(A)** Top 20 significantly enriched GO-BP terms; **(B)** Top 20 significantly enriched KEGG pathways.

After 48 h of miR-499-5p overexpression in the CPMs, the qRT-PCR results showed a highly significant increase in miR-499-5p expression compared with that in the control (*p* < 0.01, Figure 2). Four miR-499-5p overexpression and four control CPMs were collected for transcriptome sequencing. A total of 297 DEGs were detected (FDR <0.05), including 127 upregulated and 170 downregulated genes within cells overexpressing miR-499-5p compared with control cells (Supplementary Table S4). GO enrichment analysis showed that DEGs were mainly related to muscle development, muscle contraction, and muscle cell differentiation (Figure 3A). KEGG pathway analysis showed that the enriched pathways of DEGs included many muscle function-related pathways such as actin cytoskeleton regulation, focal adhesion, and cardiac muscle contraction (Figure 3B).

Furthermore, several key genes associated with muscle fiber type were found to be differentially expressed in miR-499-5p overexpressing CPMs (Table 1). Compared with our previous transcriptome data on slow- and fast-twitch chicken muscles, most of these genes shared similar expression patterns in miR-499-5p-overexpressing CPMs and those in slow-twitch muscles (Liu et al., 2020). A series of myosin genes, including *MYH1B*, *MYH1E*, and *MYH7B*, were upregulated after miR-499-5p overexpression. *MYH7B*, which encode slow myofibrillar proteins, was highly expressed in the overexpressed cells (log2foldchange = 2.42). The *MYH1B*, *MYH1E*, and *MYH7B* expression patterns were confirmed using qRT-PCR (Figure 2).

**TABLE 1:** Expression change of myofiber type-related DEGs between miR-499-5p overexpressing and NC CPMs. Trends in gene expression changes between the present study and our previous study on slow-twitch and fast-twitch muscles in chickens are also shown (Liu et al., 2020).

Gene symbol	log_2_(FC) in miR-499-5p vs. NC CPM	log_2_(FC) in slow vs. fast muscles	FDR	Expression change trends between studies
*AKAP6*	−0.37	2.08	5.7E-06	Inconsistent
*ATP2A2*	0.50	6.14	3.85E-43	Consistent
*CAMKK2*	−0.49	1.35	0.0002	Inconsistent
*CAPN3*	−0.56	−2.30	7.7E-07	Consistent
*CAV1*	0.28	1.75	3.97E-06	Consistent
*CAV3*	−0.27	−2.25	0.0001	Consistent
*CSRP3*	−0.34	−1.40	1.68E-09	Consistent
*FGF10*	0.28	1.01	5.83E-06	Consistent
*FILIP1*	−0.33	−10.46	3.97E-06	Consistent
*ITGA4*	0.26	1.09	0.0012	Consistent
*KLHL40*	−0.35	−1.20	3.16E-07	Consistent
*KLHL41*	−0.53	1.10	1.05E-37	Inconsistent
*MYBPC1*	0.68	10.50	5.97E-26	Consistent
*MYH1A*	1.09	5.36	1.12E-27	Consistent
*MYH1B*	1.29	5.18	2.1E-104	Consistent
*MYH1C*	0.85	2.12	5.42E-31	Consistent
*MYH1E*	0.62	−7.87	1.07E-11	Inconsistent
*MYH1F*	0.79	1.81	6.87E-21	Consistent
*MYH1G*	1.08	7.13	2.13E-48	Consistent
*MYH7B*	2.42	5.25	1.29E-11	Consistent
*MYL10*	0.66	11.25	1.12E-07	Consistent
*MYL2*	1.30	7.59	0.0014	Consistent
*MYL3*	1.27	16.86	1.22E-88	Consistent
*MYLK2*	0.42	−1.37	0.0005	Inconsistent
*MY O 7L2*	0.48	6.90	0.0002	Consistent
*MYOM2*	−0.56	−2.07	3.02E-26	Consistent
*PAK1*	0.38	1.30	0.0185	Consistent
*PITX1*	−0.34	8.99	0.0089	Inconsistent
*SOX6*	−0.83	−6.71	0.0423	Consistent
*TNNI1*	0.63	9.41	3.09E-26	Consistent
*TNNT1*	0.30	9.71	7.38E-06	Consistent
*TNNT2*	0.29	1.32	1.85E-12	Consistent
*TPM2*	0.34	2.07	5.21E-10	Consistent

### Target mining of miR-499-5p using transcriptome data

We used TargetScan and mirDB for miR-499-5p target gene prediction and obtained the target gene information with high confidence by comparing it with transcriptome data. We first identified 444 target genes of miRNA-499-5p in CPMs that could be identified as target genes using at least one tool (Supplementary Table S5). We found that the expression of more than 65% of the target genes had reduced after miRNA overexpression, with 16 reaching significant levels (FDR <0.05, Table 2). To improve the target gene prediction accuracy, we used the intersection of the results of the two tools as the possible targets of miR-499-5p. A total of 72 target genes were identified in CPMs, four of which were significantly downregulated in the overexpressed cells (FDR <0.05).

**TABLE 2:** Expression change of miR-499-5p target genes in miR-499-5p overexpressing CPMs compared with negative control. TargetScan and mirDB were used for miR-499-5p target gene prediction.

Gene symbol	Vs. TargetScan	Vs. mirDB	log_2_ (foldchange)	*P* -value	FDR
*SOX6*	yes	yes	−0.83	0.0017	0.0423
*NRIP1*	yes	yes	−0.29	3.72E-05	0.0032
*OSBPL1A*	yes	yes	−0.20	0.0016	0.0403
*PPP3CB*	yes	yes	−0.10	0.0094	0.0438
*LARP4B*	yes	no	−0.25	0.001	0.0438
*FNIP1*	yes	no	−0.17	0.0018	0.0456
*OLFML1*	no	yes	−1.11	2.98E-07	5.76E-05
*PARP9*	no	yes	−0.61	7.55E-06	0.0008
*ZNF106*	no	yes	−0.42	6.46E-15	5.28E-12
*SAMD8*	no	yes	−0.34	6.17E-06	0.0007
*NKTR*	no	yes	−0.27	0.0006	0.0292
*ESF1*	no	yes	−0.25	0.0014	0.0466
*UNKL*	no	yes	−0.24	0.0001	0.0102
*PTN*	no	yes	−0.21	0.0001	0.0093
*EGLN1*	no	yes	−0.19	8.67E-07	0.0001
*LZIC*	no	yes	−0.19	0.0001	0.0077
*FNIP2*	no	yes	−0.13	0.0077	0.0488

Among the four significantly downregulated target genes, *SOX6* showed the most significant decrease in expression (log2foldchange = -0.83). In addition, target genes associated with myofiber-type switching, including *PPP3CB*, *FNIP1*, and *FNIP2*, were identified, but they presented only lower expression changes.

### Validation of targeted binding relationship between miR-499 and target genes

**FIGURE 4:**
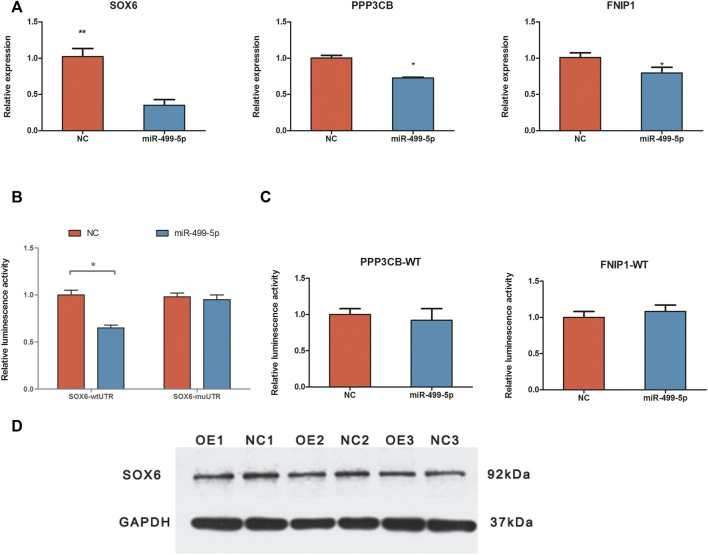
Validation of targeted binding relationship between miR-499-5p and target genes. **(A)** The relative expression levels of *SOX6*, *PPP3CB*, and *FNIP1* in miR-499-5p overexpressing CPMs and negative control by qRT-PCR; **(B)** Results of luciferase reporter assay for *SOX6* and miR-499-5p; miR-499-5p binding site mutant and wildtype vector of *SOX6*’s 3'UTR were used. **(C)** Results of the luciferase reporter as say for *PPP3CB*/miR-499-5p and *FNIP1*/miR-499-5p; only wildtype vectors were used. All results are shown as mean ± SD. **p < 0.01 and *p < 0.05 as compared with control. **(D)** The protein levels of *SOX6* in miR-499-5p overexpressing CPMs and negative control by western bolting.

We used qRT-PCR, western blotting, and luciferase reporter assay to validate the target binding relationship between miR-499-5p and the target genes (Figure 4). The qRT-PCR results revealed that the relative *SOX6*, *PPP3CB*, and *FNIP1* expression levels decreased in the overexpressed cells, with *SOX6* showing the most remarkable change in expression, consistent with the RNA-seq results. Overexpression of miR-499-5p decreased the protein level of SOX6. The luciferase reporter assay showed that miR-499-5p significantly decreased luciferase activity in combination with *SOX6* 3'-UTR sites (*p*-value < 0.05). However, the *PPP3CB* and *FNIP1* luciferase activities were not significantly altered following miR-499-5p transfection (*p*-value > 0.05).

### Effect of *SOX6* on muscle fiber specification

**FIGURE 5:**
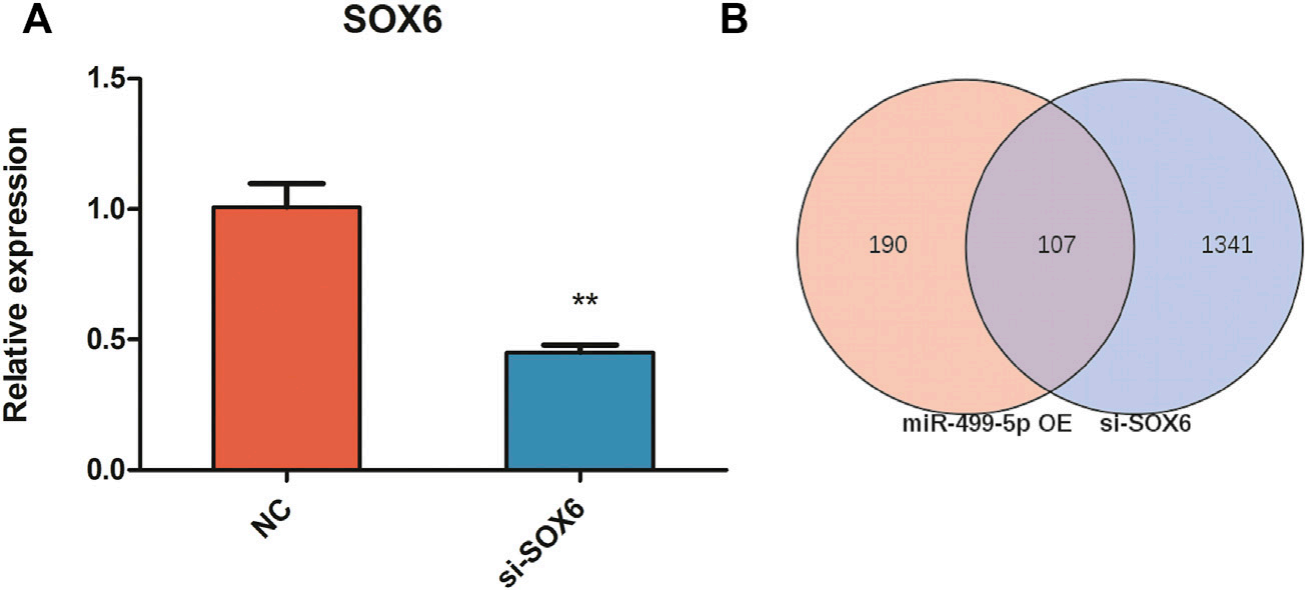
The inhibition efficiency of si-*SOX6*. **(A)** The relative expression levels of *SOX6* in *SOX6* knockdown CPMs and negative control by qRT-PCR. **(B)** The Venn diagram of differential genes in miR-499 overexpressing and *SOX6* knockdown assays. All results are shown as mean ± SD. **p < 0.01 as compared with the control.

**FIGURE 6:**
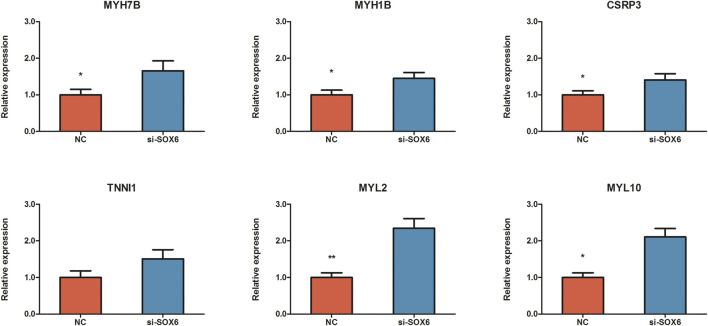
Validaton of expression change of *MYH7B*, *MYH1B*, *CSRP3*, *TNNI1*, *MYL2* and *MYL10* between *SOX6* knockdown and NC CPMs using qRT-PCR. All results are shown as mean ± SD. **p < 0.01 and *p < 0.05 as compared with control.

To investigate the effect of *SOX6* on chicken muscle fiber specification, RNA interference and RNA-seq techniques were used to obtain the expression patterns of muscle fiber-related genes and to construct a regulatory network for muscle fiber specification. After RNA interference, *SOX6* expression was 0.6- and 2.6-fold downregulated in CPMs, as determined using qRT-PCR and RNA-seq, respectively (Figure 5 and Table 3). We identified 1,448 DEGs in si-*SOX6* and control CPMs using FDR <0.05 as the screening criterion. Among these, 107 genes overlapped with the results of the miR-499-5p overexpression experiment (Supplementary Table S6). The foldchange of *MYH7B*, *MYH1B*, *CSRP3*, *TNNI1*, *MYL2* and *MYL10* expression were confirmed using qRT-PCR (Figure 6).

**TABLE 3:** Expression change of *SOX6* and myosin genes in *SOX6* knockdown CPMs compared with negative control.

Gene symbol	NC	si-*SOX6*	log_2_ (fc)	*P* -value	FDR
*SOX6*	3.36	1.27	−1.41	5.07E-07	3.08E-05
*MYH1A*	19.41	14.49	−0.42	0.0455	0.2441
*MYH1B*	198.12	329.25	0.73	0.0275	0.1227
*MYH1D*	1,123.47	509.12	−1.14	0.003	0.0234
*MYH1E*	50.04	27.38	−0.87	0.0383	0.1795
*MYH1F*	113.74	53.80	−1.08	0.04	0.1606
*MYH7*	0.50	0.75	0.59	0.0181	0.1536
*MYH7B*	0.69	1.25	0.85	0.043	0.1968
*MYH15*	835.19	422.29	−0.98	1.10E-09	2.58E-07

**FIGURE 7:**
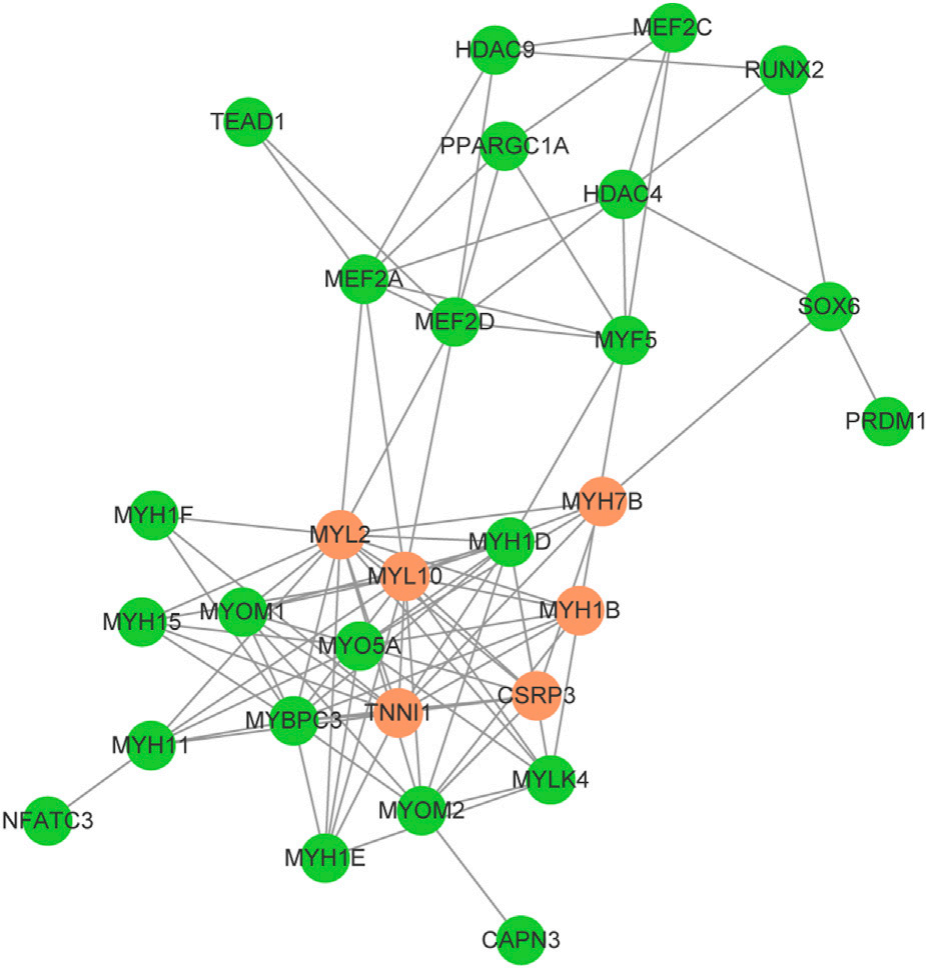
Protein-protein interaction network for the selected DEGs between *SOX6* knockdown and NC CPMs. Upregulated genes are shown in red and downregulated genes are shown in blue.

The myosin gene expression pattern revealed that the knockdown of *SOX6* resulted in significant upregulation of slow-twitch muscle-associated *MYH7*, *MYH7B*, and *MYH1B* and significant downregulation of fast-twitch muscle-associated *MYH1D* and *MYH1F* (Table 3). A total of 24 differentially expressed genes, known for myofiber type switching, were selected to build a protein-protein interaction network based on the STRING database. Among them, a total of six genes, *MYH7B*, *MYH1B*, *CSRP3*, *TNNI1*, *MYL2* and *MYL10,* were down-regulated after *SOX6* interference, while the remaining genes were up-regulated. According to the network, *SOX6* may have a direct regulatory relationship with *RUNX2*, *PRDM1*, *HDAC4*, and *MYH7B* (Figure 7).

## Discussion

Improving meat quality is the ultimate goal of broiler breeding, especially in native Chinese breeds, and the composition of muscle fibers is a critical factor affecting meat quality. For instance, high oxidative fiber level facilitates meat flavor and juiciness (Sibut et al., 2011). Muscle fiber characteristics are affected by muscle type, location, and function within an animal (Shu et al., 2017). To reveal the regulatory mechanisms affecting myofiber type in chicken skeletal muscle, we analyzed miRNA expression profiles in oxidative and glycolytic muscles in our previous study (Liu et al., 2020). miR-499-5p showed specific expression in oxidative muscles, consistent with the results observed in fish and pigs, suggesting that this miRNA is a crucial factor in regulating chicken myofiber type conversion (Duran et al., 2015; Jiang et al., 2021). In this study, a comparison of different muscles revealed a positive correlation between miR-499-5p and type I muscle fiber content and a negative correlation between miR-499-5p and type IIB muscle fiber content, implying that miR-499-5p is associated with meat quality traits.

miR-499-5p belongs to MyomiRs, which are mainly expressed in the heart and skeletal muscles and can be encoded via specific introns in slow muscle-specific myosin *MYH7B* (McCarthy, 2011). miR-499-5p positively regulates the formation of slow-twitch muscle fibers and negatively regulates the formation of fast-twitch muscle fibers in mouse and pig models (van Rooij et al., 2009; Wang et al., 2017). Although miRNAs are highly conserved among species, there is a lack of evidence regarding the effect of miR-499-5p on chicken myofiber type specification (Lee et al., 2007). In this study, RNA-seq was used to investigate the effect of miR-499-5p on the CPM transcriptome. Therefore, miR-499-5p overexpression markedly enhanced *MYH7B* and *MYH1B* mRNA expression, demonstrating its ability to promote slow-twitch muscle fiber formation in chickens. Many myofiber type-related genes were differentially expressed after overexpression and showed a similar expression pattern to that of the slow-twitch muscle. For example, cysteine and glycine-rich protein 3 (*CSRP3*), also known as muscle LIM protein (MLP), which promotes fast-twitch muscle fiber formation in chickens, showed significantly decreased mRNA expression post miR-499-5p overexpression (Shan et al., 2022). Calpain 3 (*CAPN3*) is the only muscle-specific calpain that has impotant role in muscle function such as muscle formation and remodeling (Chen et al., 2021). Studies have indicated that mutations in *CAPN3* are associated with meat quality traits including muscle fiber composion in chicken and pig (Gandolfi et al., 2011; Felício et al., 2013). In the present study, *CAPN3* was found to be downregulated following miR-499-5p overexpression, suggesting their roles in the regulation of muscle fiber types. Functional enrichment analysis revealed that DEGs are involved in several muscle development-related processes and signaling pathways associated with myofiber-type switching, including focal adhesion and regulation of the actin cytoskeleton (Ohnuki et al., 2013; Graham et al., 2015). Collectively, miR-499-5p promotes slow-twitch muscle fiber formation through diverse regulatory factors and signaling pathways.

miRNAs are involved in regulating biological processes by binding to the 3'UTR of target mRNA to repress gene expression (Bartel, 2009). Transcriptome data and bioinformatic tools were used to identify miR-499-5p target genes. More than 60% of the predicted target genes showed decreased expression in the CPM, indicating that the miRNA transfection in this study was effective. Under stringent prediction criteria, *SOX6* was identified as the target gene with the highest decrease in expression. *SOX6* is a transcription factor that has a critical effect on embryonic muscle development and myofiber maintenance (Hagiwara et al., 2005; Hagiwara et al., 2007; Quiat et al., 2011; Jackson et al., 2015). In chickens, copy number variation in *SOX6* is associated with muscle development and body weight (Lin et al., 2018; Fernandes et al., 2021). Previous studies have reported that *SOX6* is a target of miR-499-5p in skeletal and cardiac muscles (Jia et al., 2016; Wang et al., 2017). Our results revealed opposing expression trends of miR-499-5p and *SOX6* in different chicken skeletal muscles, consistent with the results observed in Nile tilapia and pigs (Nachtigall et al., 2015; Wang et al., 2017). The luciferase reporter assay showed that *SOX6* is the direct target of miR-499-5p, which may regulate chicken myofiber specification by controlling *SOX6* expression. Other genes, including *FNIP1* and *THRAP1*, have also been reported to be important targets of miR-499-5p related to muscle fiber-type specification (Liu et al., 2016; Xu et al., 2018). *THRAP1* did not show a significant decrease in expression after miR-499-5p overexpression, and *FNIP1* was not significantly associated with miR-499-5p. This study revealed that *SOX6* is the primary target of miR-499-5p in chicken myofiber-type conversion.

To further understand the effect of *SOX6* on chicken myofiber type specification and the regulatory network involved, we performed a transcriptome analysis of *SOX6*-knockdown CPMs. As expected, the *SOX6* knockdown resulted in the formation of slow-twitch muscle fibers and a decrease in fast-twitch muscle fibers. Protein-protein interaction network analysis revealed that *SOX6* could act directly on myosin and functional genes related to muscle fiber type. An et al. found that *SOX6* could directly target and bind *MYH7B* and inhibit its expression using CHIP-seq analysis, and the same result was obtained in the present study (An et al., 2011). Although fast-twitch muscle myosin genes, such as *MYH1D* and *MYH1F*, were significantly reduced after *SOX6* knockdown, there was no evidence that *SOX6* could act directly on them. *RUNX2* is a critical transcription factor, and *SOX6* and *RUNX2* interact during osteogenesis (Zhang et al., 2015). *RUNX2* has not been determined to play a role in the specification of muscle fiber types; combined with our findings, we hypothesize that *SOX6* and *RUNX2* can initiate muscle fiber type-related signaling pathways such as the PGC1α/MEF2C pathway (Shan et al., 2020).

In conclusion, we first investigated the role of miR-499-5p and *SOX6* in regulating myofiber type in chicken skeletal muscle using RNA-seq. The results revealed that both miR-499-5p and *SOX6* enhanced slow-twitch muscle fiber formation and that *SOX6* is the primary target of miR-499-5p in this process. This study provides detailed transcriptome sequencing results and serves as a reference for related studies on chicken meat quality and other species.

## Data Availability

The datasets presented in this study can be found in online repositories. The names of the repository/repositories and accession number(s) can be found below: https://ngdc.cncb.ac.cn/gsa, CRA007272.
